# All surgical supra-annular aortic valvar tissue prostheses are labelled too large

**DOI:** 10.1093/icvts/ivad076

**Published:** 2023-05-15

**Authors:** Astrid Gerritje Maria van Boxtel, Massimo Alessandro Mariani, Tjark Ebels

**Affiliations:** Department of Cardio-Thoracic Surgery, University Medical Centre Groningen, Groningen, the Netherlands; Department of Cardio-Thoracic Surgery, University Medical Centre Groningen, Groningen, the Netherlands; Department of Cardio-Thoracic Surgery, University Medical Centre Groningen, Groningen, the Netherlands

**Keywords:** Valve prosthesis, Valve labelling, Valve size, Aortic valve prosthesis

## Abstract

**OBJECTIVES:**

Surgical supra-annular aortic valvar tissue prostheses are labelled in an inconsistent and confusing manner. If the replaced valve is too small for a given patient, the risk of patient-prosthesis mismatch is increased, which is associated with the risk of morbidity and mortality. The labelled diameter (LD) of these valves should coincide with the inflow orifice diameter (IOD). Therefore, our goal was to measure all relevant IODs.

**METHODS:**

Valvar design was assessed in terms of the intended position of the valve in relation to the patient’s annulus. The IODs of all available supra-annular aortic valvar prostheses were measured using a conical gauge. The IODs were compared to the LDs. We searched for instructions for use, websites, packing boxes and regulatory institutions involved in the process.

**RESULTS:**

Eight valve models from 4 manufacturers were included. None of these valves were clearly labelled as supra-annular on the packing box, although for 3, the supra-annular label could be found in the written specifications. All valves had an IOD smaller than their LD, with a median difference of 15% (range: 4%–25%). The departure from LD differed per valve model and valve size.

**CONCLUSIONS:**

Valve packages should be labelled accurately and clearly so that surgeons can make a well-informed choice. Currently essential information is missing because the intended position in relation to the annulus is not consistently marked on the packing boxes, and valve sizes are labelled incorrectly. We propose a change for the better: relabel all valves according to their true IOD in a structured manner.

**Figure:**
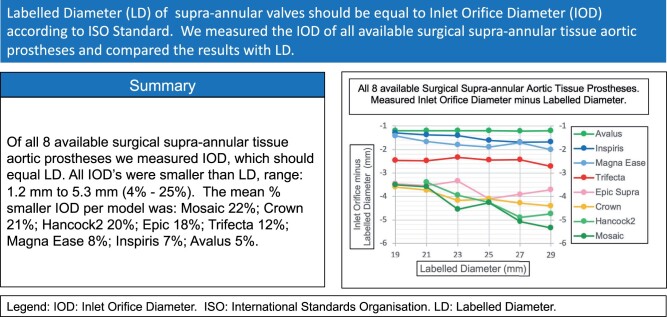

## INTRODUCTION

The labelled diameter (LD) of a cardiac surgical prosthetic valve describes a metric of the valve that is meant to coincide with the patient’s annular diameter (PAD). The LD is printed on the packing boxes for these prostheses and should reflect the PAD in full millimetres. However, in practice, the LD is presented in a confusing manner and is often larger than the PAD.

Diameter labelling, among all other aspects of surgical aortic valvar prostheses, is standardized by the International Organization for Standardization (ISO) and has been codified in Standard #5840 since 1984. The ISO is a federation of national standards institutes, with headquarters in Geneva, Switzerland (www.iso.org). These standards are created by ISO working groups, largely populated by representatives of the manufacturers, employees of national standards bodies and a few surgeons; no members of associations of cardiac surgeon are included. The standards have been updated every 5 to 10 years, resulting in a total of 6 editions. In 2015, the ISO started numbering #5840 anew because a third part was added to transcatheter inserted valve prostheses. This change led to considerable confusion. Enforcement of the standards is the responsibility of regulatory organizations such as the U.S. Food and Drug Administration (FDA) in the United States and the so-called Notified Bodies in the European Union (EU).

Nonetheless, surgeons themselves are barely aware of the existence of this standard. At the same time, manufacturers are largely responsible for the content of the standard because surgeons regard size-labelling irregularities fatalistically and consider them to be an inconvenient fact of cardiac surgical life. Yet, surgeons function with an implicit mental construct regarding fitting LDs of various valvar models into patients with given bodily characteristics and corresponding haemodynamic needs.

**Figure 1:**
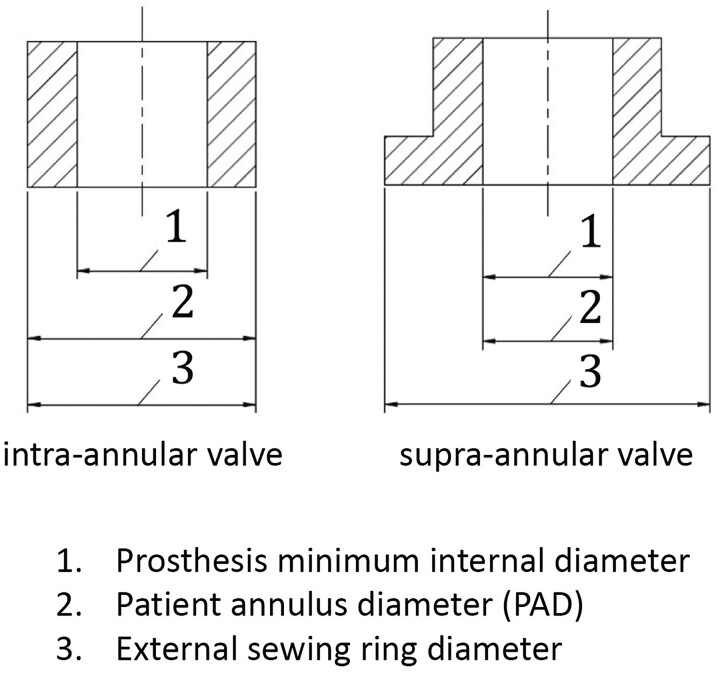
Differences between intra- and supra-annular valves. Designation of the dimensions of surgical heart valve substitute sewing ring configurations from the ISO publication: ISO 5840-2:2021(E). The information has been published with the permission of NEN, Delft NL, on behalf of ISO.

Patient-prosthesis mismatch (PPM) defines the situation when the prosthesis is too small for a given patient, resulting in a relevant gradient [1, 2]. ISO Standard #5840 defines 2 types of surgical aortic valve prostheses: one type designed for intra-annular and the other for supra-annular insertion (Fig. 1). Although valvar prostheses were designed originally for *intra*-annular insertion only, *supra*-annular prostheses have gained universal acceptance because of their superior haemodynamic performance [3], the reason being that none of the space within the patient’s annulus is occupied by parts of the prosthetic valve, unlike the situation with the intra-annular prostheses. Because supra-annular valves can be attached on top of instead of within the patient’s annulus, the prosthetic inflow orifice diameter (IOD) can and should equal the PAD, according to ISO#5840. Yet the literature suggests that among the myriad of surgical valve prostheses, various discrepancies exist between the IOD and the LD. Nonetheless, beginning with its 2005 edition, ISO#5840 specifically states that the LD should equal the IOD in supra-annular prosthetic valves [4].

Thus, even though ISO Standard #5840 has been adopted worldwide, the labelling of the diameter of prosthetic valves is far from uniform among manufacturers and valvar models, which is a pitfall for surgeons and can lead to the inappropriate choice of a prosthetic valve or an operative strategy and subsequent PPM [5–12]. A joint workforce of the American Association for Thoracic Surgery (AATS), the European Association for Cardio-Thoracic Surgery (EACTS) and the Society of Thoracic Surgeons (STS) published 2 papers recommending changes in information available to surgeons on characteristics of individual valvar prostheses [13, 14]. However, despite the fact that these publications, which appeared in 2019 and 2021, emphasized the importance of IOD, nothing has changed in LD practices. Nonetheless, knowing the actual IOD of each valve is essential for selection of an adequately sized valve and for comparison of various valvar prosthetic models, but there is as yet no published systematic, comprehensive and independent investigation into actual IODs.

We screened manufacturer-supplied information on the prosthetic designs relative to the intended intra- or supra-annular position according to ISO#5840. Then, we measured the IODs of all currently available supra-annular stented tissue aortic valve prostheses and compared the IODs with their LDs. This article provides the first comprehensive overview of the way these bioprosthetic aortic valves, largely available in Europe and North America, are currently labelled and how diameter labelling could and should be improved to reflect IOD accurately and enable fair comparisons of valves. Thus, the goal of this paper was the comprehensive measurement of the LD, which concerns the physical fit of the prosthesis in relation to the PAD, not its haemodynamic properties.

## METHODS

### Ethics statement

Because no patients or animals were involved in this study, we did not request ethics approval.

### Data availability

All relevant data are within the manuscript and its supporting information files.

We identified all commercially available supra-annular stented aortic valvar tissue prostheses. We investigated ISO#5840 meticulously as to the rules applicable to diameter labelling. We studied valvar features that would qualify the valves as supra- or intra-annular as described in ISO#5840–2:2021(E) [3]. The document specifies in article 3.7: “*intra-annular: wholly or partially within the patient’s annulus*” and in article 3.8: “*supra-annular: region wholly above the patient’s annulus*”. In article 6.3.3, the labelling shall include the *“intended position in relation to the annulus”.*

All supra-annular models of tissue aortic valve prostheses were collected, both porcine aortic and bovine pericardial valves. Valves in our archive past their expiration date for sterilization were the primary source for our investigation. In addition, manufacturers were contacted to request sample valves. Furthermore, we studied their instructions for use (IFU) and the manufacturer websites to find supplied information on the intended use and the IOD.

**Figure 2:**
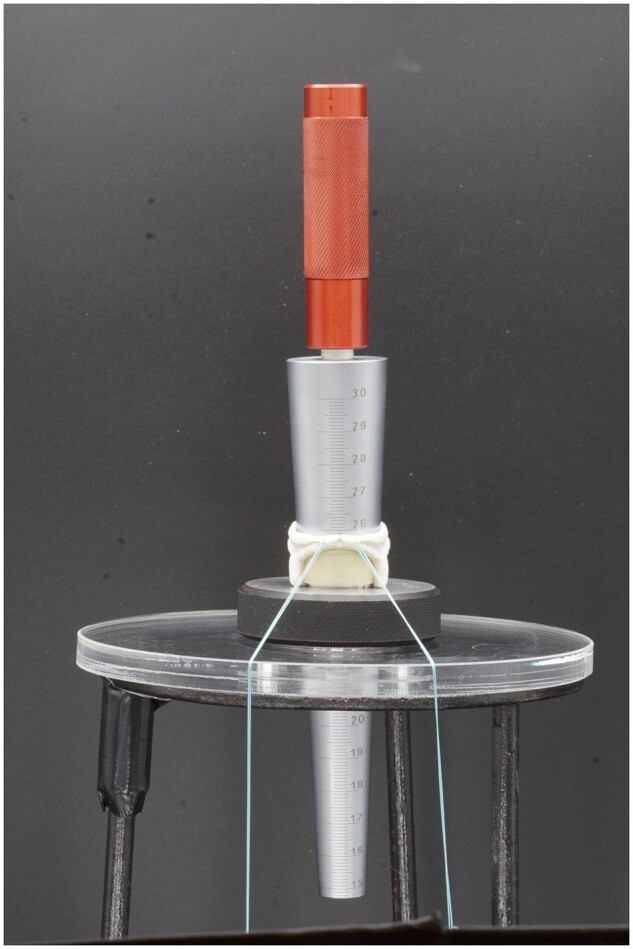
Study set-up to measure inflow orifice diameter with a standardized conical sizer. The green sutures pull down the sewing ring to reveal the inflow orifice.

The protocol for measuring the IOD included a conical gauge with a range from 15 mm to 30 mm, with a spatial resolution of 0.1 mm (Schut Geometrical Metrology, Duinkerkenstraat 21, 9723BN Groningen, The Netherlands; www.schut.com). The conal angle of the sizer is 5.7 degrees, its weight is 634 grams (Fig. 2). The ISO is currently in the process of publishing a so-called Publicly Available Specification [ISO/PAS 7020:2023: Sizing surgical valve prostheses: Guidance on the application of ISO#5840–2 (https://www.iso.org/standard/82572.html)] describing this methodology, which (at the time of this writing) has been published on 17 May 2023. All valves were poised standing with their struts on a setting ring. The valves were kept wet with saline to keep friction as uniform and low as possible.

Each sequence began with 2 mock measurements to allow for tissue compression, if any, within the inflow orifice. The magnitude of the tissue compression was analysed. Thereafter, 2 separate observers (AvB and TE) independently performed 3 measurements each. The means and standard deviations of the measurements were calculated. Intra- and interobserver variability were analysed using the Bland-Altman analysis.

Rounding of the mean IOD measurement resulted in an “expected label” in full millimetres (<0.5 down and ≥ 0.5 up). The LD was compared with the measured IOD, and the difference and percentage deviation were calculated.

When manufacturers stated that valves could be implanted in either a supra-annular or an intra-annular position, they were considered supra-annular valves, according to the ISO#5840–2:2021(E) article 3.8 rule: “*wholly above the patient’s annulus”*. If the geometric orifice area was provided in the IFU of the prosthesis, this metric was calculated back to the corresponding diameter.

Additionally, we investigated which regulatory authorities are involved in certifying valvar prostheses in different countries and for specific manufacturers and models.

## RESULTS

**Figure 3:**
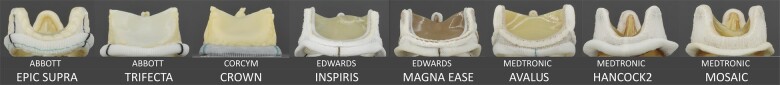
Studied surgical supra-annular aortic tissue valves, arranged in alphabetical order by manufacturer and model name.

After observing the ISO criteria for supra-annular valves, we included the following valvar prostheses in our investigation: Abbott Epic (Biocor) Supra and Trifecta (Abbott Vascular, Santa Clara, CA, USA); Corcym Crown (Corcym, Burnaby, BC, Canada); Edwards Inspiris and Magna Ease (Edwards Lifesciences, Irvine, CA, USA); and Medtronic Avalus, Hancock2 and Mosaic (Medtronic, Minneapolis, MN, USA) (Fig. 3). Of these valves, 4 were introduced to the market after the 2005 ISO Standard Edition, which presented the rules for valve labelling: Epic, Trifecta, Avalus and Inspirus. The Epic, Hancock2 and Mosaic are aortic porcine valves; all the other valves are constructed from bovine pericardial tissue.

The intended position in relation to the PAD, such as “supra-annular”, was not printed on any of the packing boxes for the prosthetic valves. However, in their IFUs, the 2 Abbott valves and the Medtronic Avalus valve were correctly designated as supra-annular. The IFUs for all the other valves indicated that both the supra- and intra-annular positions were possible. Nonetheless, because no part of these valves protrudes through the annulus when it is implanted supra-annularly, the latter is the correct designation according to the ISO#5840. We had 8 models from 4 manufacturers to investigate. Each of the 8 models are produced in 6 LDs from 19 mm through 29 mm at intervals of 2 mm; even numbers do not exist. Only the Medtronic Hancock2 valve, diameter 19 mm, was no longer marketed or available. We were able to collect 44 of the 47 valves (94%) of the 8 existing models. The expected IODs of the 3 missing valves were extrapolated by linear regression. Printed information is becoming less easily available, except for the IFU, which is usually hidden within the sealed packing box in a non-sterile condition. Some companies list the inner diameter of the internal semi-rigid stent on their website; however, these websites are apparently revised regularly and are thus not always consistent. Obviously, a stent diameter measured without the mounted valvar tissue is thus the diameter of an unfinished product. Medtronic specifications are the only ones readily available online; Abbott, Corcym and Edwards specifications are currently impossible to find through the companies’ websites, in contrast to previous versions of their websites. The collected specifications by the manufacturers, currently online or not, are downloadable through the link provided with this publication. For Corcym, this is the specification from its predecessor, LivaNova.

**Figure 4:**
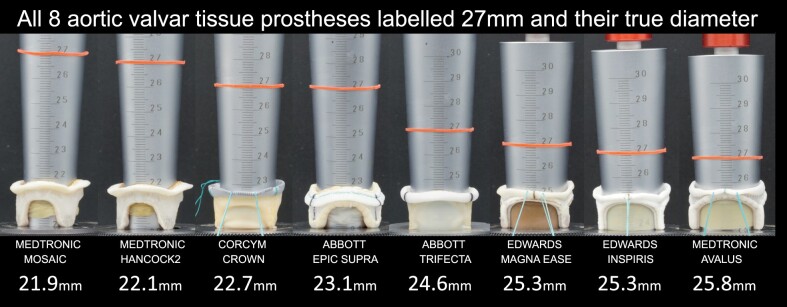
Test results of all 27-mm labelled valves. Measurements with the conical gauge of the internal orifice diameters of surgical aortic tissue valves labelled 27 mm, arranged sequentially from largest to smallest differences between labelled and measured sizes. The red rubber band is at the 27.0 mm grade mark. Labels indicate manufacturers and model names of the valves and their measured inflow orifice diameters.

The IODs measured according to our protocol produced the mean measured IODs reported and illustrated in Table 1. The differences between the LDs and the measured IODs in all valves labelled 27 mm are illustrated in Fig. 4. We chose the 27- mm LD prostheses because it was one of the 3 LD sequences that was complete. Tissue compression during the initial 2 mock measurements was slightly greater in the porcine aortic valves compared to that in the bovine pericardial valves. The range of interobserver difference was 0–0.2 mm (absolute mean: 0.04 mm, SD: 0.06 mm). Intra-observer variability for observer 1 had a range 0–0.15 mm (mean: 0.03 mm, SD: 0.03 mm); for observer 2, range 0–0.21 mm (mean: 0.03 mm, SD: 0.04 mm). There were no relevant differences between the observers.

**Table 1:** Differences between labelled and measured valve sizes

Labelled size (mm)	Inflow orifice diameter (mm)	Correct labelled size (mm)	Label oversized by percent
**Abbott Epic Supra**			
19	*15.9*	*16*	*20%*
21	*17.7*	*18*	*18%*
23	19.7	20	17%
25	21.4	21	17%
27	23.1	23	17%
29	25.3	25	15%
**Abbott Trifecta**			
19	16.5	17	15%
21	18.5	19	13%
23	20.7	21	11%
25	22.6	23	11%
27	24.6	25	10%
29	26.3	26	10%
**Corcym Crown**			
19	15.4	15	23%
21	17.2	17	22%
23	19.0	19	21%
25	20.9	21	20%
27	22.7	23	19%
29	24.5	25	18%
**Edwards Inspiris**			
19	17.7	18	7%
21	19.6	20	7%
23	21.6	22	6%
25	23.4	23	7%
27	25.4	25	6%
29	27.3	27	7%
**Edwards Magna Ease**			
19	17.6	18	8%
21	19.3	19	9%
23	21.6	22	7%
25	23.4	23	7%
27	25.3	25	7%
29	27.0	27	7%
**Medtronic Avalus**			
19	17.8	18	7%
21	19.8	20	6%
23	21.8	22	6%
25	23.8	24	5%
27	25.8	26	5%
29	27.8	28	4%
**Medtronic Hancock2**			
**19**	N/A	N/A	N/A
21	17.6	18	19%
23	19.1	19	21%
25	20.8	21	20%
27	22.1	22	22%
29	24.3	24	20%
**Medtronic Mosaic**			
19	15.5	15	23%
21	17.4	17	21%
23	18.5	18	25%
25	20.7	21	21%
27	21.9	22	23%
29	*23.7*	*24*	*25%*

Measurements of the inflow orifice diameter (IOD) per valve brand, model and labelled size (LS) are shown in black. Estimated values for 3 missing valves are derived by linear regression and are depicted in *green italics*. The correct LS is derived by rounding off the measured and extrapolated values to full millimetres. The percentage of oversizing by the LS is depicted in the last column.

None of the valves have an LD that equals their measured IOD, with a mean difference of 2.8 mm (SD: 1.3 mm; range: 1.2 mm–5.3 mm). There were substantial differences between the models (Table 1; Central Image). The image illustrates that the differences between the IOD and the LD are fairly linear for the pericardial valves. However, for the porcine aortic valves, the smaller diameters show less difference between the IOD and the LD than the larger diameters. There seem to be step-ups in the curves for the Hancock2 and Mosaic, but somewhat less so in the Epic Supra (Central Image).

The authorities involved included the U.S. FDA (www.fda.gov; www.accessdata.fda.gov). In Europe, 24 Notified Bodies from 11 countries are recognized for the certification of cardiac valve prostheses (EU Medical Device Regulation 2017/745). Under European law, Notified Bodies are private companies licensed to conduct the certifying process for products to obtain the CE mark with which these products obtain access to the EU market. The notified bodies involved are for Abbott: BSI-NL, CE2797, BSI Group, The Netherlands B.V. (www.bsigroup.com); Corcym: the U.S. FDA; this valve is no longer registered in the EU; Edwards: DEKRA, CE0344, DEKRA Certification B.V., Arnhem, The Netherlands. (www.dekra-product-safety.com); Medtronic: BSI-NL and TÜV-SÜD: CE0123, TÜV SÜD Product Service GmbH Zerzifizierstellen, München, Germany (www.tuev-sued.de/ps). Since Brexit, the Medical Healthcare Products Regulatory Agency (www.gov.uk/government/organisations/medicines-and-healthcare-products-regulatory-agency) instead of the notified bodies has been responsible in the UK since 1 January 2021.

## DISCUSSION

All observed IODs were smaller than the corresponding LDs that were found in the IFUs and/or on the packing boxes, ranging from 1 to 5 mm over all makes and diameters. Therefore, all valves need to be relabelled, because the surgeons are and have been put on the wrong foot by the implicit suggestion that the prosthetic IODs are larger than they actually are. Due to these inaccurate LDs, surgeons cannot easily compare valvar models in relation to their IODs. Yet the IOD is exceedingly important because it determines the largest valve that can physically fit onto a patient’s annulus. When the annulus per se is too small, the surgeon can consider enlarging it surgically to prevent PPM. Ideally, surgeons should know in advance what the minimal acceptable PAD=IOD is for a specific patient. Current valve labelling results in a smoke screen prohibiting surgeons from seeing the actual relevant information. Relabelling is a one-time effort that has to be accompanied by worldwide re-education of all involved health-care professionals. Thereafter, we can concentrate on the haemodynamics and durability on a level playing field.

LD and inflow “naked” stent diameter as listed in some of the IFUs have been taken into consideration in previous studies, but nobody except Bapat [15, 16] verified the IOD dimensions of the finished product. Previous studies comparing valve size to results are erroneously influenced by the usage of the manufacturer-supplied dimensions, which, as we know now, do not reflect the IOD [12, 15]. Only the Crown IFU contains a column with near perfect IOD values but these were, paradoxically, not used for diameter labelling. The Inspiris IFU contains a column listing geometric orifice areas, which, after calculating back to the corresponding IOD, coincide well with our IOD measurements. We can only conclude that manufacturers are aware of the IOD but have not used it for their labelling despite the ISO Standards. Paradoxically the ISO working group writing the Standard is dominated by these very manufacturers.

In contrast to supra-annular prostheses, intra-annular prostheses should be labelled according to the diameter of the intra-annular part of the valve, starting with the 2005 edition of ISO#5840, because the leading principle is that prosthetic valvar labels should reflect the PAD. Thus, the design of the prostheses intended for supra- or intra-annular placement is of paramount importance and should be clearly stated according to the 2021 edition of ISO#5840 (Rule 6.3.3). Since 2005, a total of 2 updated ISO#5840 editions have been published, 1 in 2015 and 1 in 2021; in neither case has the definition essentially changed.

The manufacturers actually never fully implemented the 2005 ISO#5840 Standard, both as to diameter labelling and as to design intentions. At the same time, regulatory authorities such as the U.S. FDA, the Medical Healthcare Products Regulatory Agency and the European Notified Bodies have not enforced this ISO Standard. At least the U.S. FDA was fully aware of this long-standing problem as early as 2003 and advised “*that selection of a satisfactory device fit be confirmed by testing the prosthesis within the annulus before insertion of sewing cuff sutures* before the publication of the first edition, which included this definition of LD [17]. None of the Notified Bodies has ever disclosed anything on this issue because of the confidential relationship they have with their clients, the manufacturers.

In 2017, the AATS, the EACTS and the STS decided to install a joint task force to advise surgeons on the packaging boxes and the labelling. However, nonconflicted surgeons were a minority of the task force members, and there was a high degree of overlap between the ISO Working Group and the intersocietal task force. Differing constitutions, formats and focus complicated this initiative. Eventually, the 2 papers that were published did not advise relabelling [13, 14]. Instead, standardized EOA charts were advised, which are based on the results of clinical postoperative echocardiography studies. Their implementation is controversial because they are based on clinical data from various clinical cohorts and are therefore unreliable [14]. The fact that manufacturers participated in writing the standards, both in the ISO working group and in the AATS/EACTS/STS Task Force, might have played a role in this delay of the standardization. Therefore, the ISO Standard has been ineffective in regulating valve labelling, despite the fact that the regulators were also part of the working group.

The manufacturers have pointed out that new editions of the ISO Standard apply only to “*both newly developed and modified heart valve substitutes and to the accessory devices, packaging and labelling required for their implantation and for determining the appropriate diameter of heart valve substitute to be implanted”* (article 1.2 ISO#5840:2005). This article remained essentially unchanged in the subsequent editions. However, as can be read on the publicly available U.S. FDA website (www.accessdata.fda.gov), many valve models were introduced after 2005, and all existing valves went through multiple “modifications” without adaption of the labelling to the Standard. In addition, the following provision was introduced in the 2015 edition: “*Labelling of prosthetic cardiac valves that have been on the market before the current International Standard version shall be adapted to conform to current standards”* (article C1 ISO#5840:2015).

Prevention of PPM remains a paramount argument for updating diameter labelling to the true IOD of supra-annular valves. The manufacturers have not articulated any argument in favour of keeping the labelling as it is. The rumoured reason that surgeons would be confused by relabelling valves is presumptuous and degrading. On the contrary, surgeons are currently confused by the incorrect labelling.

Multiple previous publications have indicated that valve diameter labelling is chaotic. Bonchek complained about this issue as early as 1987, when the first 1984 ISO Standard was published, but did not specify a particular methodology regarding sizing [8]. Christakis described the problem in 1998 in response to the publication of the third edition of ISO#5840 in 1996 [9]. His comments probably resulted in the recommendations of the next edition in 2005, with consent of these very manufacturers, that thereafter did not change the labelling, while the regulators followed suit. Youdelman wrote in 2007 in his introduction: “*Labeled valve diameter is not the same as millimetre measure of the prosthetic valve diameter or the aortic annulus into which it will fit”,* apparently totally unaware of ISO#5840, as ostensibly were the reviewers [4, 10]. The mere existence of an ISO Standard for cardiac valves seems to have been obscure, and current general awareness is probably similar. Eichinger complained that “*manufacturers’ valve diameters are misleading because of specific differences in geometric dimensions”* [18, 19]. Bapat then investigated in 2013 the internal diameter of tissue valves using Hegar dilators, specifically to facilitate catheter-bound valve-in-valve procedures [15]. While using Hegar dilators, he used excessive, but unspecified, force to replicate the forces employed during these procedures. In addition, we have noticed that Hegar dilators are subjected to considerable frictional forces with the leaflets and are thus less suitable for measuring the IOD [16]. Neither the Avalus nor the Inspiris was measured by Bapat because they were introduced after Bapat's publication. The differences between our measurements and those of Bapat varied per model: smaller valves were generally measured larger by us.

Because we found differences between manufacturers and models, we call for relabelling of all supra-annular aortic tissue valvar prostheses, as indicated in Table 1. LivaNova, Corcym’s predecessor, chose not to renew their CE certification in 2019; hence this valve is no longer on the European market but is available elsewhere.

In summary, we showed that supra-annular aortic valvar prostheses are all labelled too large. None of the valves are labelled in accordance with the applicable ISO Standard. Only after relabelling will surgeons appreciate the true IOD that has to fit to the PAD in these types of valves. Most manufacturers are resistant to change the labelling, and professional organizations have added to the confusion by advocating clinical reference charts derived from various methodologies and studies instead of just sticking to the physical metrics. Just like with all medical implants and materials, factual measurements such as a diameter should be verifiably correct. Current diameter labelling may have added to the problem of PPM but to what extent is not known.

### Limitations

The limitations of this article are that we only measured surgical stented supra-annular aortic valves and that 3 valve diameters were missing, which prompted us to use extrapolation to calculate the probable IOD.

## CONCLUSION

Current valve labelling is misleading and can have harmful consequences for patients. Therefore, it is essential that valve labelling and information on valve packages be accurate and clear. This article shows the extent of the problem of unclear labelling. We demonstrate the need for a change and recommend relabelling of supra-annular aortic valvar prostheses to their intended position in relation to the annulus and the true IOD, as our measurements have shown.

## Data Availability

All relevant data are within the manuscript and its supporting information files.

## Glossary

AATS: American Association for Thoracic Surgery
EU: European Union
FDA: Food and Drug Administration
IFU: Instructions for use
IOD: Inflow orifice diameter
ISO: International Organization for Standardization
LD: Labelled diameter
PAD: Patient annulus diameter
PPM: Patient-prosthesis mismatch
STS: Society of Thoracic Surgeons
